# Impact of prenatal life on the risk of developing epilepsy

**DOI:** 10.3892/mi.2024.136

**Published:** 2024-02-08

**Authors:** Nina Otinashvili, Saba Ahmadi, Luka Iordanishvili, Anashwara Balagopal, Tsotne Gvasalia

**Affiliations:** American MD Program, Faculty of Medicine, Tbilisi State Medical University, 0177 Tbilisi, Georgia

**Keywords:** epilepsy, prematurity, premature birth, birthweight, gestational age

## Abstract

Epilepsy is an enduring predisposition of the brain to generate epileptic seizures and has a worldwide incidence of 21-24 per 100,000 cases among children. Epilepsy is a multifactorial disease; however, certain risk factors are predicted to increase its incidence. Abnormal brain development during prenatal life, particularly during the last trimester, is considered to play a crucial role in the development of certain neurological disorders. The present study evaluated a total of 453 children between the ages of 1 to 18 years, with or without epilepsy. The association between gestational age, birth weight, maternal age and sex, and the risk of developing epilepsy was examined in the children. It was found that children born preterm had a 2.3-fold higher risk of having epilepsy [odds ratio (OR), 2.3; 95% confidence interval (CI), 1.4-3.7], and those whose birth weight was <2,500 g had a 2-fold greater risk of developing epilepsy (OR, 2; 95% CI, 1.1-3.6). The male sex appeared to be associated with a lower risk of developing epilepsy and there was a statistically significant association between the female sex and the risk of developing epilepsy only in preterm children (OR, 3.2; 95% CI, 1.2-8.8). Maternal age was not found to be associated with the risk of developing epilepsy. On the whole, the present study demonstrates that a short gestational age, a low birth weight and the female sex are associated with an increased risk of developing epilepsy.

## Introduction

The International League Against Epilepsy (ILAE) defines epilepsy as a disorder of the brain characterized by an enduring predisposition to generate epileptic seizures (1). The worldwide incidence of epilepsy among children aged 11-17 years is 21-24 per 100,000 cases. Epilepsies are multifactorial disorders with a poorly understood etiology that are considered to be caused by an array of genes and environmental factors (2). Abnormal brain development during prenatal life is considered to play a crucial role in certain neurological disorders with a clinical onset later in life. Neuroimaging technology has further emphasized this point (3). Preterm delivery and a low birth weight have been found to be associated with an increased risk of developing cerebral palsy (4), cognitive delay (5) and behavioral disorder (6).

A growing body of evidence has revealed associations between preterm birth and an increased risk of developing epilepsy in early life (7). The link between the risk of developing epilepsy and post-term birth, however, has not yet been elucidated. Ehrenstein *et al* (8) demonstrated an increased risk of developing epilepsy with prolonged gestation. However, this may be attributed to instrument-assisted and cesarean deliveries that can cause complications at birth (8).

A nationwide cohort in Finland revealed a decreased risk of developing epilepsy with an increased gestational age, with the cumulative incidence of epilepsy in the cohort being 0.54% (9). From both a clinical and public health perspective, it is important to identify such risk factors. This identification prompts the question of whether these factors could be modifiable through alterations in obstetric practices.

Birth weight, a factor often influenced by gestational age, has been found to be lower in patients with epilepsy compared to healthy controls (10). The effect of sex on the development of childhood epilepsy has been controversial. While some studies have found that males have a higher tendency to develop this condition (11), others have found no significant difference (12).

The correlation between maternal age and a number of neuropsychiatric diseases has been studied. An extreme maternal age range appears to be a risk factor (13,14). A young maternal age, and specifically teenage motherhood, may contribute to socioeconomic disadvantages and in numerous diseases, including epilepsy, there appears to be a social gradient suggesting that children born to parents of a lower socioeconomic position face an elevated risk of developing the disease (15). In addition, an advanced maternal age has been linked to increasing offspring disease risk due to higher rates of genomic alterations and a higher risk of perinatal and obstetric complications (16). The present study aimed to investigate these variables in relation to epilepsy. To the best of our knowledge, variables such as birth weight, maternal age and sex have been negligibly explored in previous studies (8,9).

## Patients and methods

### Patient information

A case-control study was conducted on 154 patients with epilepsy and 299 controls. The study was approved by the Ethics Committee of Iashvili Children's Central Hospital (a university hospital of Tbilisi State Medical University), Tbilisi, Georgia (approval no. N 22-0929-1311a). Signed informed consent was collected from the parents/legal guardians of each study participant. The inclusion criteria were an age range of 1-18 years and the diagnosis of epilepsy. All patients were recruited from two hospitals (Iashvili Children's Central Hospital and Medison Clinic) in Tbilisi, Georgia. The controls were children in the same age range, who were hospitalized without having been diagnosed with epilepsy. The exclusion criteria included children who had severe chronic or multisystemic health conditions, severe developmental delays, or any other neurological conditions. Information such as gestational age, birth weight, maternal age and sex were collected from patients' medical records. Patient seizures were classified based on ILAE 2017 as generalized, focal and unspecified onset seizures (17).

### Statistical analysis

All statistical analyses were performed using GraphPad Prism 9.3.1 for macOS (Dotmatics). Data were analyzed using the Chi-Squared and Fisher's exact tests, and odds ratios (ORs) and 95% confidence intervals (CIs) were calculated.. All statistical tests were planned to be two-sided. A value of P<0.05 was considered to indicate a statistically significant difference.

## Results

To examine the association between gestational age and the risk of developing epilepsy, the participants were categorized into the pre-term (<37 weeks of gestation), term (37-41 weeks) and post-term groups (>41 weeks). There was a statistically significant difference between the gestational ages of the cases and controls (Chi-square test value, 11.84; P=0.0027). Compared with children whose gestational age at birth was 37-41 weeks, children with a gestational age <37 weeks had an almost 2-fold greater risk of having epilepsy (OR, 2.3; 95% CI, 1.4-3.7). Post-term birth was not associated with an increased risk of developing epilepsy compared to the term children (Table I).

The male sex was found to be associated with a lower risk of developing epilepsy compared to the female sex (OR, 0.6; 95% CI, 0.4-0.9). However, a maternal age >35 years was not found to be a significant risk factor (OR, 1.0; 95% CI, 0.5-1.8) (Table I).

**Figure 1:**
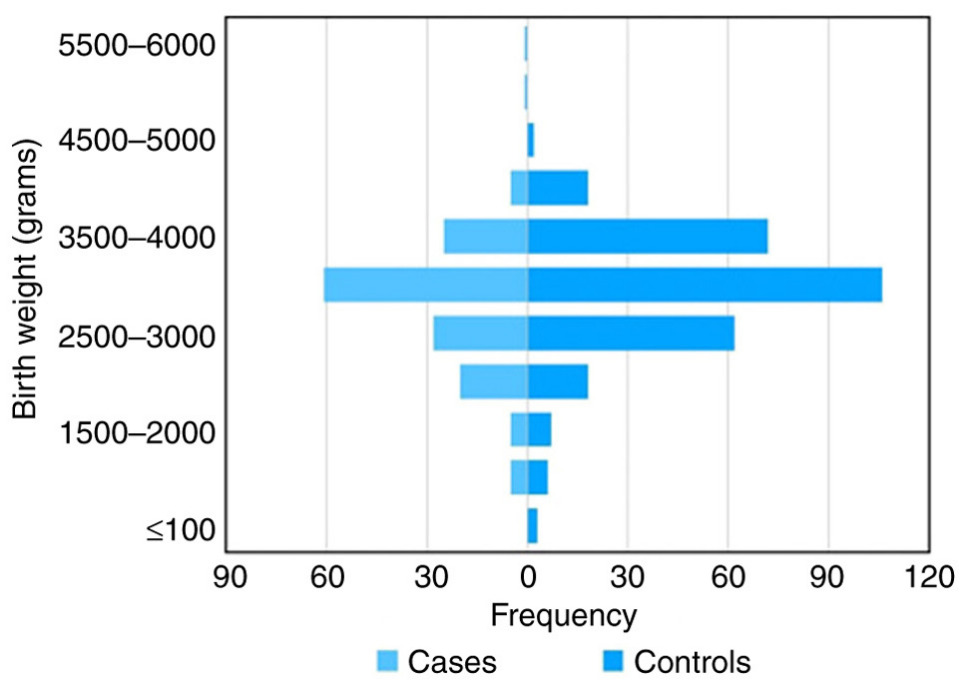
Distribution of birth weight. The graph shows the distribution of birth weight in the epilepsy cases and the controls.

The distribution of birthweight in the study population is presented in Fig. 1. Compared with the children whose birth weight was >2,500 g, those whose birth weight was <2,500 g had a 2-fold greater risk of developing epilepsy (OR, 2; 95% CI, 1.1-3.6). The multiple risk factors that were studied in association with epilepsy are presented in Table I.

**Figure 2:**
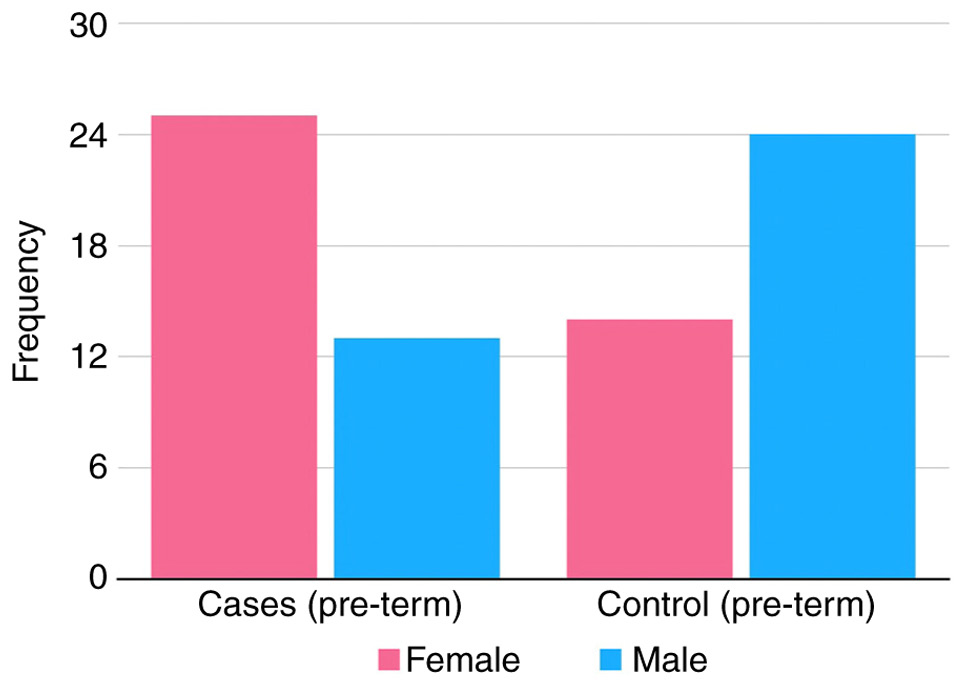
Sex distribution in the pre-term cases and controls. The graph demonstrates the frequency of females and males in the pre-term epilepsy cases and pre-term controls with normal development.

The proportion of male and female patients in the pre-term epilepsy cases compared to the proportion of male and female patients in the pre-term controls was also investigated (Fig. 2). There was a statistically significant association (P=0.0211) between the female sex and the risk of developing epilepsy only in pre-term children (OR, 3.2; 95% CI, 1.2-8.8). The risk of a pre-term female child being diagnosed with epilepsy was ~3.2-fold that of a pre-term male child being diagnosed with epilepsy (Table II).

In addition, the association between the type of seizures and the study variables, including gestational age, sex, maternal age and birth weight was examined (Table III). It was found that there was a statistically significant difference between the type of seizure and gestational age; however, lager studies with higher sample numbers are required for a definitive conclusion.

## Discussion

The present study identified an increased risk of epilepsy in children who were born prematurely, before 37 weeks of gestation, compared to children born full term. Arpino *et al* (18) conducted a study on neonatal seizures in the first week of life and found that neonatal seizures were strongly associated with a low gestational age and a low birth weight, two related factors that can predispose neonates to seizures. They found that the key etiology of neonatal seizures was hypoxic-ischemic encephalopathy (30%), which can be mediated by a low birth weight and gestational age (18). In the present study, the risk of developing epilepsy in pre-term births was >2-fold higher (OR, 2.3) than that in term births. Brain volume markedly increases during the last trimester, with a 4-fold increase in gray matter and a 5-fold increase in white matter (19). Premature birth can increase the risk of developing epilepsy, as the last trimester is a critical time for the brain development of the fetus (20). In addition, premature birth is often accompanied by other risk factors during pregnancy, such as infection and preeclampsia (21,22). Prenatal stress can affect the fetus via multiple mechanisms in the long term. In addition to increasing preterm birth and low birth weight, prenatal stress can affect the central nervous system of the fetus by stress hormones released from the body of the pregnant mother. Neurotransmitters in the brain can be disturbed by being exposed to stress hormones, such as glucocorticoids and corticotrophin-releasing hormones. There is supporting literature on the general association between stress in the early stages of life and seizures (23). Considering the adverse effects of preterm gestational age and prenatal stress on the chances of developing epilepsy, the multidisciplinary management of pregnant women needs to be implemented in order to reduce preterm birth and stress. Ehrenstein *et al* demonstrated that prolonged gestation may be a risk factor for epilepsy (8); however, in the present study, this could not be confirmed with the data obtained.

Another variable in the present study was birth weight, specifically a birth weight <2,500 g. After analyzing the data, it was observed that patients born with a birth weight <2,500 g were 2-fold more likely to develop epilepsy. This may be due to the fact that the premature brain is more susceptible to seizures (7). Febrile seizures are also more common in preterm children and those with a low birthweight (24).

In the present study, there was a significant associated risk between developing epilepsy and the female sex. According to the results obtained, the risk of a preterm female child being diagnosed with epilepsy was ~3.3-fold greater than that of a preterm male child being diagnosed with epilepsy. Following the analysis of all the subgroups together, females were found to be more likely to develop epilepsy. Epidemiological research has suggested that boys are more likely than girls to develop febrile seizures, and are 1.5-2-fold more likely to develop multifocal epileptic syndromes (25). However, idiopathic generalized epilepsy is more frequent among females. The reason behind these differences is not fully understood; however, it may be related to sex hormones, as they directly influence the development of the mammalian brain and modulate brain activity (11,12). Since sex remains controversial in association with the development of epilepsy, further, more extensive and diverse research in a similar vein is warranted to elucidate this matter. An advanced maternal and paternal age has been recognized to affect the risk of developing a number of congenital diseases, including neuropsychological disorders (26). For example, a significantly increased risk of developing psychosis has been found in the offspring of mothers at an advanced age (27). However, no significant difference was found in the risk of developing epilepsy between the offspring of mothers >35 years and <35 years of age in the population in the present study.

The present study has certain limitations which should be mentioned. The study was limited by a lack of detailed clinical data on the types of epilepsy, limitation of size and follow-up. There are more factors to be considered as variables in future research, such as the form of delivery (natural vs. cesarean sections) or complicated birth (with or without the assistance of instruments).

In conclusion, the findings of the present study demonstrate that gestational age at birth and birth weight are associated with a subsequent risk of developing epilepsy. Environmental factors during fetal development or a shorter gestation period could potentially contribute to the developing of epilepsy, particularly in young children.

## Figures and Tables

**Table I:** Risk of developing epilepsy according to gestational age, sex, maternal age and birth weight.

Parameter	Cases, n (%)	Controls, n (%)	OR (95% CI)	OR P-value	P-value^a^
Gestational age					
Pre-term	38 (24.6%)	38 (12.7%)	2.3 (1.4-3.7)	0.0011^b^	
Term	110 (71.4%)	254 (84.9%)	1.00 Ref		
Post-term	6 (3.9%)	7 (2.3%)	1.9 (0.6-5.4)	0.2293	0.0027^b^ (χ^2^=11.84)
Sex					
Male	74 (48.0%)	178 (59.5%)	0.6 (0.4-0.9)	0.0202^b^	
Female	80 (51.9%)	121 (40.4%)	1.00 Ref		0.0218^b^
Maternal age, years					
>35	20 (41.6%)	114 (40.8%)	1.0 (0.5-1.8)	0.9164	
<35	28 (58.3%)	165 (59.1)	1.00 Ref		>0.9999
Birth weight, kg					
<2.5	23 (15.2%)	24 (8.1%)	2.0 (1.1-3.6)	0.0243^b^	
>2.5	128 (84.7%)	269 (91.8%)	1.00 Ref		0.0332^b^

^a^P-value was calculated from the Chi-squared test for gestational age and Fisher's exact test was used for sex, maternal age and birth weight;

^b^statistically significant difference (P<0.05). OR, odds ratio; CI, confidence interval.

**Table II:** Risk of epilepsy according to sex in pre-term cases and controls.

Sex	Pre-term cases, n (%)	Pre-term controls, n (%)	OR (95% CI)	P-value
Female	25 (65.7%)	14 (36.8%)	3.2 (1.2-8.8)	0.0211^a^
Male	13 (34.2%)	24 (63.1%)	1.00 Ref	

^a^Indicates a statistically significant difference. OR, odds ratio.

**Table III:** Association between the type of seizure and gestational age, sex, maternal age and birth weight.

Parameter	Generalized onset seizure, n (%)	Focal onset seizure, n (%)	Unspecified onset seizure, n (%)	P value^a^
Gestational age				<0.0001^b^
Pre-term	25 (80.6%)	22 (27.5%)	10 (27.0%)	
Term	6 (19.3%)	58 (72.5%)	27 (72.9%)	
Sex				0.1
Male	15 (46.8%)	46 (54.7%)	13 (34.2%)	
Female	17 (53.1%)	38 (45.2%)	25 (65.8%)	
Maternal age, years				0.5
>35	4 (33.3%)	5 (55.5%)	12 (44.4%)	
<35	8 (66.6%)	4 (44.4%)	15 (55.5%)	
Birth weight, kg				0.6
<2.5	5 (15.6%)	14 (16.6%)	4 (10.5%)	
>2.5	27 (84.3%)	70 (83.3%)	34 (89.4%)	

^a^P-value calculated from the Fisher's exact test;

^b^statistically significant difference (P<0.05).

## Data Availability

The datasets used and/or analyzed during the current study are available from the corresponding author on reasonable request.
